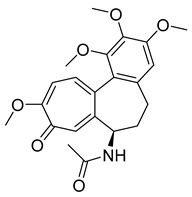# Small-Molecule Mitotic Inhibitors as Anticancer Agents: Discovery, Classification, Mechanisms of Action, and Clinical Trials

**DOI:** 10.3390/ijms26073279

**Published:** 2025-04-01

**Authors:** Yazmin Salinas, Subhash C. Chauhan, Debasish Bandyopadhyay

**Affiliations:** 1 School of Integrative Biological and Chemical Sciences, The University of Texas Rio Grande Valley, 1201 West University Drive, Edinburg, TX 78539, USA; yazmin.salinas02@utrgv.edu; 2 Division of Cancer Immunology and Microbiology, Medicine, and Oncology Integrated Service Unit, School of Medicine, The University of Texas Rio Grande Valley, McAllen, TX 78504, USA; subhash.chauhan@utrgv.edu; 3 South Texas Center of Excellence in Cancer Research (ST-CECR), McAllen, TX 78504, USA; 4 School of Earth, Environmental, and Marine Sciences (SEEMS), The University of Texas Rio Grande Valley, 1201 West University Drive, Edinburg, TX 78539, USA

**Keywords:** small-molecule inhibitors, antibody–drug conjugates, mitosis, kinase proteins, microtubule-associated proteins, mitotic proteins, clinical trials, anticancer agent, cytotoxic, taxanes

## Abstract

Despite decades of research, cancer continues to be a disease of great concern to millions of people around the world. It has been responsible for a total of 609,820 deaths in the U.S. alone in 2023. Over the years, many drugs have been developed to remove or reduce the disease’s impact, all with varying mechanisms of action and side effects. One class of these drugs is small-molecule mitotic inhibitors. These drugs inhibit cancer cell mitosis or self-replication, impeding cell proliferation and eventually leading to cell death. In this paper, small-molecule mitotic inhibitors are discussed and classified through their discovery, underlying chemistry, and mechanism(s) of action. The binding/inhibition of microtubule-related proteins, DNA damage through the inhibition of Checkpoint Kinase 1 protein, and the inhibition of mitotic kinase proteins are discussed in terms of their anticancer activity to provide an overview of a variety of mitotic inhibitors currently commercially available or under investigation, including those in ongoing clinical trial. Clinical trials for anti-mitotic agents are discussed to track research progress, gauge current understanding, and identify possible future prospects. Additionally, antibody–drug conjugates that use mitotic inhibitors as cytotoxic payloads are discussed as possible ways of administering effective anticancer treatments with minimal toxicity.

## 1. Introduction

The American Cancer Society estimated 1,958,310 new cancer cases and 609,820 cancer deaths in the United States for the year 2023. Additionally, it was found that incidence numbers for breast, prostate, and uterine corpus have been on the rise. Although these numbers appear daunting, it is important to note that the cancer death rate decreased by 1.5% between the years 2019 and 2020. Compared to data from 1991, this indicates an estimated 3.8 million lives were saved [1]. The steadily decreasing cancer mortality rate can be largely attributed to increasingly advanced cancer treatments. Thus, to further combat the increasing cancer incidence affecting men and a significantly large percentage of women, discovering and enhancing cancer treatments is of great interest. One of many currently studied treatments is small-molecule mitotic inhibitors. Mitotic inhibitors are mostly small-molecule drugs that block cell growth by stopping mitosis or cell division. To be a promising small-molecule mitotic inhibitor, a molecule should have a molecular weight of less than 900 Da with fewer than five hydrogen bond donors, ten hydrogen bond acceptors, and a calculated logP between −1 and +5. Additionally, the molecule should possess suitable stereo- and regiochemical features to bind to the target biomolecule effectively. As a result of their ability to disrupt a cancer cell’s cycle, they have been identified as anticancer agents.

A cancer cell’s growth is one of the main factors contributing to tumor growth and the prolific invasion of surrounding tissue [2]. This is because a cancer cell’s growth is often said to be ‘out of control’. The unregulated proliferation of tumor cells is caused by a cancer cell’s typically low requirements for extracellular growth factors. In some cases, cancer cells may even produce growth factors that stimulate autocrine growth [3]. Therefore, inhibiting their growth is a significant factor when combating malignant cells. One way to do so is by disrupting the cell’s mitosis, or the process by which a cell self-duplicates by equally segregating replicated chromosomes in the mother nucleus into the nuclei of two daughter cells. The process can be divided into five sub-processes: prophase, prometaphase, metaphase, anaphase, and telophase [4]. In prophase, the mitotic spindle, responsible for segregation, begins to form from microtubules and their associated proteins. Meanwhile, interphase chromatin start to condense into rod-like chromosomes as the nuclear envelope breaks down [5]. Then, in prometaphase, the mitotic spindle microtubules attach to the chromosomes, which are then lined up in the cell equator in metaphase. Sister chromatids are then split and separated onto their respective spindle poles during anaphase, and they begin to decondense as the nuclear envelope reforms during telophase [4,5]. It is worth mentioning that the explained process has been oversimplified and has redacted details on how each step is brought about. Nonetheless, it is still clear to see that microtubules and their associated proteins play a significant role throughout mitosis. This fact is exploited by small-molecule mitotic inhibitors, which sometimes target microtubulin and mitosis-associated proteins (MAPs).

Microtubule-regulating proteins are discussed in this review to emphasize their role further and lay the groundwork for the mechanism of action of many mitotic inhibitors. After that, different commercial and in-trial drugs are reviewed and categorized by natural, synthetic, and semi-synthetic, as well as their mechanism of action—microtubule binding or inhibition of microtubule-related proteins, inhibition of cell cycle checkpoint proteins causing DNA damage and non-checkpoint kinase proteins related to mitosis. Although multiple clinically studied mitotic inhibitors are discussed in this review, they do not entirely encompass all the ones that have been clinically studied. The primary purpose of this review is to provide a concise overview of the mechanistic insights into mitotic inhibitors, ranging from commercially available small-molecule drugs to molecules under investigation, and to explore possible future avenues for developing effective anticancer treatments using small-molecule mitotic inhibitors.

## 2. Microtubule Regulating Proteins

As previously stated, mitosis is a complex process that requires the coordination of a multitude of molecules and regulatory proteins [6]. This includes, most notably, microtubules and mitosis-associated kinases, which are integral to every step involved in cell replication. To understand how microtubules and kinases are targeted to prevent tumor cell mitosis and retard tumor development, the proteins and enzymes integral for successful mitosis are included in this article. The number of proteins involved in the mitotic process is quite large. The proteins discussed further have been successfully targeted for cancer treatment, and not at all a complete overview of all mitotic proteins.

### 2.1. Microtubule-Associated Proteins (MAPs) and Microtubule-Targeting Agents (MTAs)

Microtubules undergo dynamic instability, a nonequilibrium process where a subset of microtubules transition between a growing and shortening state [7]. The energy-requiring process is regulated by microtubule-associated proteins (MAPs). Some MAPs stabilize as they promote the polymerization or inhibit the depolymerization of microtubules, whereas others destabilize by increasing the frequency of ‘catastrophes’ or shrinking of microtubules [5]. Stabilizing MAPs include MAP1, MAP2, MAP4, tau, and doublecortin proteins. Tau proteins differ from MAP1, MAP2, MAP3, and MAP4 in their molecular weight as they will range below 55–63 kDa, while the others will range below 200–100 kDa. Destabilizing MAPs include oncoprotein 18, katanin, and XKCM14 [8].

Cells can regulate dynamic turnover by shifting between stabilizers and destabilizers. If MAPs do not properly regulate turnover, humans may develop a neuronal disease [7]. Additionally, since MAPs are active during mitosis, their inhibition can lead to a cell failing to replicate and eventually dying. As a result, MAPs-inhibiting small molecules have the potential to act as successful anticancer molecules and, thus, mitotic inhibitors.

Microtubule-targeting agents (MTAs) can also impact cancer apoptosis through microtubule disrupting means. MTAs can be categorized in one of two groups: microtubule-destabilizing agents or microtubule-stabilizing agents. Vinca alkaloids, conchicines, and combretastatin are classified under microtubule-destabilizing agents because they inhibit microtubule polymerization at high concentrations. On the other hand, paclitaxel, docetaxel, and the epothilones are categorized as microtubule-stabilizing agents for their ability to stimulate microtubule polymerization [9]. Regardless of the classification, pharmacologically relevant MTAs bind to β-tubulin and suppress spindle-microtubule dynamics, resulting in slower or inhibited mitosis during the metaphase/anaphase transition and eventual cell death [10].

### 2.2. Motor Proteins

Along with microtubules, motor proteins play a large role in cancer proliferation. They are active participants in a multitude of cellular functions, including organelle and chromosomal movements, intracellular transport and communication, muscle contraction, maintenance of cellular shapes and mechanical integrity, and cytokinesis. Motor proteins include the superfamilies kinesin, dynein, and myosin.

Myosin significantly differs from kinesin and dynein because it moves on actin filaments instead of microtubules. However, like the other superfamilies, myosin motor proteins convert energy from ATP to perform mechanical work along a specified track or road [11]. Additionally, myosin shares an ancestry related to GTPases with the kinesin family.

Kinesin proteins are essential for all eukaryotic cells and play a vital role in cell division. In a 2003 study, four kinesins were found to be involved in metaphase chromosome alignment and another four in bipolar spindle assembly [12]. Furthermore, they have been found to impact chromosome movement and cytokinesis. In humans, the kinesin motor protein KIFC1 (from the Kinesin-14 family), which plays a role in tumor cell division, has been observed to be significantly expressed in cancer cells of various cancers [13].

Dyneins, like kinesins, move along microtubules yet share a mechanochemical cycle similar to that of myosin [14]. Unlike myosin and kinesin, dynein evolved from an AAA+ family of motors. Regardless, they have still been observed to play an important role in cellular division as they work as the primary motor for motility along microtubules towards the end [15].

Regarding human cancer, one study investigated the importance of motor proteins in cancer by assessing copy number alterations (CNAs) and survival rates. Analysis showed that the CNAs in kinesin and dynein were substantially tied to lower survival rates [16]. This further highlights motor protein inhibitors as target anticancer treatments. For instance, kinesin inhibitors can inhibit spindle assembly or centrosome separation, leading to apoptosis. Myosin inhibitors can stabilize microtubules, stopping the cancer cells’ cycle. Similarly, dynein inhibitors can bind to cancer cells’ microtubules, stopping or retarding their growth [16].

### 2.3. Mitotic Kinase Proteins/Enzymes

**Figure 1:**
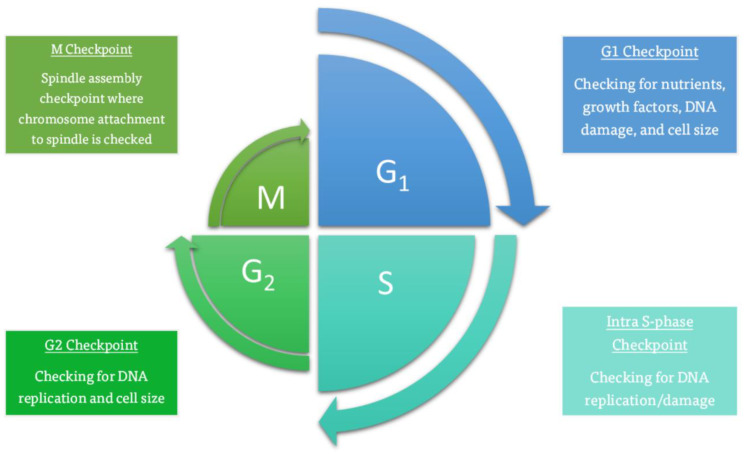
Checkpoints and cell regulation throughout the cell cycle [21].

Besides microtubule-related proteins, kinases play a crucial role in mitosis, especially regarding cell cycle checkpoints. Checkpoints regulate the steps of the cell cycle, as described in Figure 1. Their purpose is to prevent mutant cells from replicating and causing damage. At the G1 checkpoint, the cell grows, and DNA damage is checked before entering and committing to the S phase, where the DNA is to be replicated. Afterward, DNA damage is once again checked at the intra-S and G2 checkpoints. If the DNA is found to be undamaged in the G2 phase, the cell carries on toward mitosis, where the M checkpoint checks the alignment of the cell’s chromosomes and their spindle attachment.

Mutations in any checkpoint protein may result in cancer and genetic instability [17]. In terms of mitotic inhibition, the spindle checkpoint is of great interest as it detects microtubule attachment to kinetochores and inhibits transitioning from metaphase to anaphase until any detected defects have been fixed.

It has been found that mitotic kinases, such as polo-like kinase 1 (PLK1) and aurora kinases, are often overexpressed in many different cancers [18]. This makes sense as these proteins are heavily involved in cell growth; thus, their proliferation and mutation would directly affect the regulation of tumor cell proliferation [19]. This correlation is significant in that it implies that mitotic kinases have the potential to act as targets for cancer therapies that could impede cancer cell growth [20].

## 3. Mitotic Inhibitors

### 3.1. Mitotic Inhibitors Affecting Microtubulin and/or Its Related Proteins

As discussed in the previous section, microtubules and their associated proteins play a significant role in mitosis. They can often be found to be overexpressed in cancer cells. For these reasons, inhibiting and/or binding to microtubules or their related proteins can reduce and even entirely stop the growth of cancerous cells. The following sections discuss some compounds/drugs that mitotically inhibit cancer growth through microtubules (MT) and/or MT proteins. The compounds have been classified in natural, synthetic, or semi-synthetic categories. A summary of the compounds’ name, category, and corresponding structures and classifications are shown in Table 1.

#### 3.1.1. Microtubule Binding/Protein-Related Drugs: Natural

In this section, natural anticancer compounds pertaining to the binding/inhibition of MTs and MT-associated proteins are reviewed. Note that just because these compounds have natural origins, it does not mean they are administered in their natural derivation. For some compounds, such as paclitaxel, the percent composition of the anticancer agent is relatively low in its natural source, so it is substantially more cost- and time-effective to use the synthetic form of the antineoplastic drug in its place.

##### Taxanes

Taxanes are a diverse class of chemotherapy agents that act as microtubule inhibitors, inhibiting the cell cycle of cancer cells at the G2/M phase, which ultimately leads to apoptosis [23]. Taxanes include paclitaxel, docetaxel, and cabazitaxel. Since docetaxel and cabazitaxel are semi-synthetic, they will be further discussed in a later section of the article, whereas this section will mainly focus on paclitaxel.

In a 2024 study, the cytotoxic effects of taxanes were comprehensively reviewed. It was found that, although taxanes can be effective against breast, lung, esophageal, prostate, bladder, and head and neck cancers, the anticancer agents can be challenging to synthesize due to their various chiral centers. Nonetheless, extensive research has resulted in the synthesis of a handful of taxanes. Different possible mechanisms of action have been identified, including the induction of micronucleation and the activation of various caspases, which are integral to the eventual apoptosis of cancer cells [24].

Paclitaxel is a plant alkaloid isolated from the bark of the Pacific Yew tree (*Taxus brevifolia*) and the European Yew tree (*Taxus baccata*). It acts as a stabilizing microtubule-targeting agent [25]. In other words, it enhances microtubule formation, producing a stable but nonfunctional microtubule [26]. It can be used to treat breast cancer, ovarian cancer, and lung cancer, as well as AIDS-related Kaposi’s sarcoma. Its treatment may result in a wide variety of side effects, such as fever, joint and/or muscle pain, hair loss, anemia, etc. It could also cause peripheral neuropathy and anaphylaxis [26]. Therefore, it is not surprising that paclitaxel is not typically used as a first course of treatment, usually being administered after the failure of other cancer therapies.

A study in 2021 investigated the effect of paclitaxel on apoptosis and mitotic catastrophe in AGS cells [27]. It was observed that the drug activated caspase-3, caspase-9, and PARP in its mechanism of action to achieve apoptosis. Additionally, mitotic catastrophe and cell cycle arrest at the G2/M phase were observed. This could suggest that mitotic catastrophe plays a significant role in the drug’s mechanism of action. Moreover, another study on paclitaxel related to cell cycle arrest implies paclitaxel initiates apoptosis through multiple mechanisms [28]. In the end, it was concluded that the concentration of the microtubule-targeting drug is the major factor determining its apoptogenic mechanisms.

A different study took a slightly different approach, focusing more on the drug’s ability to inhibit mitotic spindle assembly and function. This was investigated by assessing paclitaxel’s ability to inhibit progression into the G1 phase. Results indicated that at low paclitaxel concentrations, the formation of mitotic spindles was inhibited without affecting function or arresting cells in mitosis [29].

Not only have taxanes been extensively studied for their cytotoxicity, but they have also been investigated for their synergetic effect when used with other active agents. One example of this is its synergetic relationship with YM-01. YM-01 plays a significant role in arresting cancer cell growth by inhibiting a class of chaperones called heat shock protein 70 (HSP 70) and potentially reducing Tau protein levels in vitro and ex vivo. This inhibition is of great interest because HSP 70 aids cancer cells in resisting therapeutic agents by protecting them from physiological, pathological, environmental, and pharmacological disturbances [30]. Thus, by inhibiting HSP 70 with YM-01, paclitaxel may synergize effective treatment. A handful of studies on taxanes and their synergetic application with other drugs have been conducted. However, the main focus of this review is the anticancer potential of individual mitotic inhibitors. For this reason, synergetic relationships between active agents and mitotic inhibitors are not discussed in further detail. Nonetheless, it is important to be at least aware of the possibility of synergistic applications with small mitotic inhibitors as they are a popular study of interest.

##### Vinblastine and Vincristine

Vinblastine and vincristine are vinca alkaloids isolated from the leaves of the Madagascar periwinkle plant, *Catharanthus roseus*, and *Vinca rosea*. Like other vinca alkaloids, they belong to a class of phase M-specific anti-tubulin agents [31]. By binding to tubulin, vinca alkaloids inhibit the formation of microtubules, which are essential for mitosis. Therefore, through their binding, vinblastine and vincristine can cause metaphase arrest [26]. This mode of action contrasts with that of taxanes. Where the taxanes act as stabilizers, vinca alkaloids like vinblastine work as destabilizers [25].

Vinca alkaloids have similar mechanisms of action but significantly differ in antitumor spectrum, dose, and clinical toxicity. Vinblastine can be used to treat Hodgkin’s disease, certain lymphomas, breast cancer, uterine cancer, testicular cancer, and Kaposi’s sarcoma. Often, it is not administered on its own but in combination with other cancer drugs. The drug’s side effects may include low blood cell counts, mouth pain or ulcers, nausea, etc. As a result, people with very low white blood cell counts and who are prone to bacterial infection should not be treated with vinblastine. Some similar side effects may also arise with the use of vincristine. However, unlike vinblastine, vincristine can be used to treat rhabdomyosarcoma, neuroblastoma, and Wilm’s tumors [32].

##### Epothilones

Epothilones are a macrolide class of anticancer agents with anti-tubulin activity. Initially, they were found to be produced as secondary metabolites of the myxobacterium *Sorangium cellulosum* [33]. In-depth investigations on their bioactivity revealed that these molecules could cause tubulin polymerization and induce apoptosis and cell cycle arrest at G2/M through stabilization. Epothilones are significantly helpful in fighting against cancer cells that have become resistant to taxane chemotherapy. For their substantial anticancer activity, epothilones continue to be studied and their synthetic and semi-synthetic derivatives continue to be synthesized. Unfortunately, most developed derivatives fail in either Phase II or Phase III clinical trials. Only the derivatives ixabepilone and utidelone are used in clinical practice.

In more recent years, however, new epothilone derivatives with even better activity against refractory tumors than ixabepilone or utidelone have emerged with the development of synthetic strategies. It is possible that with further research these derivatives can be developed into clinically approved anticancer drugs [34].

##### Combretastatin A4

Combretastatin is a class of natural phenols—stilbenes (combretastatin A), dihydrostilbenes (combretastatin B), phenanthrenes (combretastatin C), and macrocyclic lactones (combretastatin D)—found in the bark of the South African Bush Willow tree (*Combretum caffrum*) [35]. Combretastatin can cause rapid collapse of a tumor’s vascular structure by targeting VE-cadherin and β-catenin/AKT signaling pathways. Additionally, they have antioxidant, anti-inflammatory, and antimicrobial activity.

Out of all the combretastatins, combretastatin A4 is currently the most tested in preclinical and clinical trials as it exhibits the highest anticancer activity in this class [36]. CA-4 is able to prevent αβ-tubulin dimer assembly and cause eventual cell death by binding to the β-tubulin binding site, colchicine. The effect of CA-4 inhibitors has been studied against human bladder cancer cells and in a murine orthotopic bladder tumor model [37]. The results showed that CA-4 induced G2/M phase arrest and caspase-3 activation in cancer cells. It was also observed that intravesical CA-4 therapy slowed down the growth of murine bladder tumors.

CA-4P, a prodrug of CA-4, passed clinical trials and offered several advantages, including better water solubility, longer circulation, better drug targeting, and lower toxicity [36]. However, it has been discontinued due to unfavorable partial response data and a lack of significant improvement in progression-free survival [38].

Moreover, various CA-4 analogs have been studied between the years 2016 and 2023. From these studies, analogues with azetidine-z-one, pyrazole, sulfide, or carrying a selenium atom were observed to have significant anticancer activity through different pathways. Analogues with 3,4-diaryl pyrazole were effective against breast, ovarian, cervical, and colorectal cancer. The organoselenium analogue worked well against breast, ovarian, cervical, and hepatic cancers. β-lactam derivatives and sulfide-bridged CA-4 analogues were toxic to breast cancer. Therefore, for their substantial cytotoxic activity, further research on CA-4 analogues would be promising avenues of interest. They provide varied ways of cell apoptosis with fewer side effects [39].

##### Trichostatin A

Trichostatin A (TSA) is a natural derivative of dienohydroxamic acid containing antifungal properties. It was originally obtained from a culture broth of *Streptomyces platensis*. Research has found that TSA can act as a potent histone deacetylase (HDAC) inhibitor [40]. In a 2001 study, the antiproliferation and HDAC inhibitory activity of TSA was assessed using in vitro human breast cancer cell lines and an in vivo carcinogen-induced rat mammary cancer model. A total of eight breast carcinoma cell lines were tested, and similar HDAC inhibitory activity was observed in all of them. In regard to the rat mammary cancer model, it was found that TSA had significant anticancer activity by comparing the cancer proliferation of 16 treated animals and 14 untreated control animals [41].

Furthermore, another study done in 2005 compared cell cycle progression of the spindle checkpoint with TSA treatment on human cancer cells alone versus TSA combined with nocodazole or paclitaxel [42]. When 200 ng/mL of TSA (only) was administered for eight hours, class I and II HDACs were successfully blocked, but the progression into mitosis was not interrupted. The formation of the mitotic spindle continued; however, there was a missegregation of chromosomes, which is considered a hallmark of cancer. When TSA was administered with nocodazole or paclitaxel, TSA blocked the phosphorylation of BubR1, a mitotic checkpoint, which resulted in rapid cell death. These results imply that TSA can increase the effectiveness of microtubule-disrupting drugs such as nocodazole and paclitaxel. Further research on TSA’s synergetic properties might be useful, so this is certainly a point to look back on.

According to recent research, the cytotoxic activity of TSA is not limited to its strong link to the inhibition of HDAC. In fact, the agent’s mechanism of action is varied and full of multiple possible pathways, based on the type of cancer being treated.

In regard to brain cancer, TSA inhibits the proliferation of glioblastoma (GBM) by the upregulation of p21^WAF1^ and p53 and downregulation of cell cycle regulators CDK4 and 6. In neuroblastoma, TSA inhibits cell viability by promoting the acetylation of Ku70. Against tongue squamous cell carcinoma, the cytotoxic agent induced apoptosis through the downregulation of hypoxia-inducible factor-1α protein and vascular endothelial growth factor (VEGF). Thus, with all the possible pathways to cancer cell death brought about by TSA, it is strongly encouraged to further investigate the agent’s extensive anticancer properties as a possible anticancer drug [43].

##### Nordihydroguaiaretic Acid (NDGA)

Nordihydroguaiaretic acid (NDGA) is a phenolic lignan isolated from the creosote bush of Mexican and U.S. deserts, also known as the *Larrea tridentata* [44]. It is a highly bioactive compound used over time to treat several diseases, including cancer, cardiovascular/immunological/neurological disorders, and even aging.

In vitro, NDGA (1–100 μΜ) has been effective against various tumors and leukemia cell lines. Its concentration and type of tumor cells play a significant role in its mechanism of action. One such way is the inhibition of lipoxygenase metabolites, which can act as growth-promoting factors in many cancer cells [45]. In a 2007 study, it was proven that NDGA induces the accumulation of microtubule dynein-dynactin complex at the centrosome. Additionally, it is observed that in mitotic cells, NDGA induced dynein-dynactin accumulation at the spindle poles [46]. This relationship could mean that NDGA could act as a good mitotic inhibitor.

##### Colchicine

Colchicine is a plant-based alkaloid that can be extracted from *Colchicum autumnale* and *Gloriosa superba*. Initially, it was used to treat gout and amyloidosis [47]. However, it was later discovered to act as a small-molecule mitotic inhibitor. The first studies on the compound’s effect on mitosis can be traced back to 1937 [48]. Various research has established colchicine disrupts cell motility, intracellular movement, cell polarity, and mitosis [49]. Its effects are particularly apparent in tissues involving a high mitotic rate, like those in bone marrow.

To assess the drug’s mechanism of action on mitosis of human cells, we look at a study done using H3-colchicine. It was observed that colchicine concentration and cell binding were proportional, as expected. Binding also appeared to be reversible. After six to eight hours of exposure to 100 nM of colchicine, it was found that all cells were blocked in metaphase, suggesting that the drug bonded to most of the interphase cells. In other words, the drug can cause mitotic arrest of cancer cells by inhibiting the polymerization of tubulin into microtubules and preventing the formation of the mitotic spindle [50].

#### 3.1.2. Synthetic Microtubulin Binding/Protein-Related Drugs

In this section, synthetic drugs that target microtubules or MT-related proteins are discussed.

##### Nocodazole

The drug nocodazole acts as a mitotic inhibitor by highly specific binding to the plus ends of microtubules. Nocodazole binds to β-tubulin, disrupting microtubule dynamics and, thus, the formation of spindles during the metaphase part of mitosis. As a result, the G2/M phase is arrested, and apoptosis in tumor cells occurs [51].

##### Monastrol

Monastrol is a small-molecule inhibitor that can pass the cell membrane and inhibit the motor protein kinesin-5, which plays a significant role in spindle bipolarity and mitosis. In recent years, monastrol and monastrol analogs MA-1 and MA-2 (Table 1) have been increasingly developed to produce effective anticancer agents with limited side effects or toxicity. One such study reported that monastrol is a possible option for cancer therapy, resulting in little toxic effects on neurons. The article investigated how monastrol affected exposed cultured sympathetic neurons, especially when compared to taxol, another commonly used anticancer therapy [52]. Results showed that monastrol was noticeably less toxic to the neurons over time and could be a good way of treating cancer without sacrificing a patient’s neuronal health.

Furthermore, a series of dihydropyrimidine (DHPM) derivatives containing a 1,3,4-oxadiazole moiety were synthesized as monastrol analogs and screened for potential anticancer activity toward 60 cancer cell lines [53]. Analysis of HL-60(TB) cells treated with MA-1 and MOLT-4 cells treated with MA-2 exhibited G2/M arrest and apoptotic activity. Thus, the drug had good anticancer activity. Another study revealed the antiproliferation activity of monastrol analogs enastron and dimethylenastron; however, rather than structure, it focused on their effects against glioblastoma cells in a kinetic crystal violet assay. Additionally, the vasastrol VS-83 analog was also investigated. The drugs showed higher antiproliferation activity against U-87 MG, U-118 MG, and U-373 MG glioblastoma cells [54]. When investigated for p-glycoprotein 170 activity, the compounds were observed to have zero modulation effects on p-gp. This implies that the tested monastrol analogs are potential anticancer drugs and might also be less neurotoxic than other classic tubule inhibitors.

##### S-Trityl-L-Cysteine

Like monastrol and its analogs, S-Trityl-L-Cysteine (STLC), a synthetic derivative of the cysteine α-amino acid, inhibits the microtubule motor protein involved in mitotic pole separation, kinesin-5. Studies have shown that STLC is one of the most potent in vitro and in cell-based assays [55].

To further understand the inhibition mechanism of STLC, a study investigated the drug’s effect in HeLa and U2OS cells and in vitro. Results showed that after a longer incubation time, HeLa cells with STLC had a G2/M block that persisted for up to 72 h. On the other hand, prolonged incubation of U2OS cells resulted in adaptation and mitotic slippage, after which the cells could continue to a second round of DNA replication [49]. After reviewing all the data from the investigation, it was found that STLC blocks the cell cycle at mitosis—not anywhere near S and G2 progression. The drug also did not interfere with microtubule distribution. Instead, STLC inhibited centrosome separation, which resulted in a monoastral spindle and eventually mitotic arrest because of the activation of the spindle assembly checkpoint [55].

Furthermore, a different study on STLC investigated the drug’s cell cycle arrest in terms of dosage and treatment of neuroblastoma, one of the most common types of solid tumors affecting young children [56]. It was found that the arresting quality of STLC was dependent on its concentration and that the anticancer treatment successfully demonstrated cell apoptosis and cell cycle arrest in neuroblastoma cancer cell lines. With all that being said, STLC appears to be a good mitotic inhibitor against cancer. However, it is important to note that the drug’s zwitterion structure complicates its clinical development because it causes solubility issues. Masking its two free terminal amino and carboxyl groups also proves difficult because they play an integral role in binding kinesin-5. Therefore, developing STLC-derived compounds that can overcome these solubility issues is worth exploring further [57].

##### Dynarrestin

While some drugs, like STLC, inhibit kinesin motor proteins, other drugs have the potential to inhibit dynein motor proteins. An example is dynarrestin, a novel dynein inhibitor capable of inhibiting cytoplasmic dynein 1 and 2 [16]. Dynarrestin is of special interest because of its distinct specificity.

Dynarrestin contributes to several cellular functions, such as hedgehog (Hh) signaling and mitosis. The Hh pathway has been observed to contribute to the growth of various cancers. Therefore, treatments to inhibit this pathway have been developed. However, the success rates of these drugs are not as high as one would hope because currently available Hh pathway inhibitors also target Smoothened (Smo), which can acquire certain mutations that improve the cancer’s resistance to cancer treatment [58]. Hence, Hh inhibiting treatments are not currently sufficient, as they also need to be able to do so without targeting Smo. In a 2016 study, multiple small molecules, including dynarrestin, were screened to find a possible solution to the Hh pathway-Smo dilemma. It was discovered that dynarrestin potently blocked Hh signaling and, consequently, the proliferation of tumor cells dependent on Hh. Additionally, and most remarkably, the drug did not bind with Smo or SUFU. Furthermore, it was observed that dynarrestin reversibly interfered with proper mitotic spindle orientation and dynein-motor protein-based microtubule motility in vitro without any significant blocking of ATP hydrolysis [58]. Therefore, the study showed that dynarrestin can function well as a good mitotic inhibitor with distinct specificity. This success implies that other dynein-inhibiting or dynein-targeting molecules can have similar results in combating different cancers or even controlling Hh signaling without interacting with Smo.

##### Blebbistatin and Its Derivatives

Blebbistatin is a small-molecule mitotic inhibitor with a high affinity for non-muscle myosin II, which plays an essential role in cell division and motility. It binds to the ATPase intermediate and slows down phosphate release. While it does not interfere with myosin binding to the actin filament, it does block myosin heads through low-actin-affinity product complexes [59].

In terms of muscle physiology and the exploration of the cellular function of myosin II isoforms, blebbistatin can be useful. However, its applicability is minimal because of its poor water solubility, high fluorescence, cytotoxicity, and inclination to (photo)degradation [60]. To compensate for this, we need to look further at blebbistatin derivatives that could allow for the specific inhibition of myosin II while not being limited by so many factors.

#### 3.1.3. Semi-Synthetic Microtubulin Binding/Protein-Related Drugs

The following drugs are analogs or 2nd/3rd metabolites from natural compounds with mitotic inhibitory activity targeting microtubules/tubules and/or MT-related proteins.

##### Docetaxel

Like paclitaxel, docetaxel can be used to treat mainly breast and lung cancer. Moreover, it can also treat prostate cancer, stomach cancer, and head/neck cancer. It is typically administered through injection, and, similar to paclitaxel, may cause a list of side effects, including sudden vision problems, redness or swelling in arms or legs, skin rashes, muscle fatigue, swelling, confusion, loss of appetite, low blood cell counts, and even liver problems. For these reasons, someone with liver disease, a low white blood cell count, or non-small cell lung cancer should not be considered for its treatment.

Despite its multiple side effects, docetaxel is still used to combat various cancers, including chemo-resistant prostate cancers. The anticancer agent works by binding to β-tubulin and preventing depolymerization [61]. Moreover, some studies have found that docetaxel can possibly trigger anticancer immune cells, which are capable of killing malignant cells [62].

In phase II studies, docetaxel was found to have a prostate-specific anticancer response rate of 42%. When administered in combination with estramustine (Emcyt), results suggest a better response than docetaxel itself. Therefore, docetaxel is not only a good small-molecule inhibitor but also capable of synergizing the therapeutic effect when combined with appropriate biological response modifiers [63].

Other studies have honed in on the effect of docetaxel on human breast cancer cells. In one such study, the cellular responses to the drug were assessed by evaluating breast cancer cell viability, cell cycle checkpoint arrest, and death mechanisms [64]. It was concluded that the drug’s primary mechanism of action was mitotic catastrophe.

Another study sought to increase the drug’s mitotic arrest. It underlined how breast cancer patients who are treated with docetaxel chemotherapy often experience poor outcomes and may undergo relapse, so further attempts must be made to improve results against breast cancer. One such attempt involved using the Bcl-2-associated death promoter (BAD) as a prognostic indicator for successful or positive docetaxel treatment [65]. Results suggested that tumors that were BAD-expressive were more susceptible to taxane treatment. By investigating the cellular mechanism of taxane on BAD-expressing xenograft tumors, it was found that the drug caused the cells to have lengthened mitotic arrest and an increasing number of deaths during mitosis. The deaths were found to be necroptotic and ROS-dependent rather than non-apoptotic and non-Bcl-XL-dependent. This implies that BAD is a good prognostic for favorable treatment with taxane chemotherapy.

##### Cabazitaxel

Cabazitaxel is a semi-synthetic antineoplastic agent in the taxane class (10-deacetylbaccatin III) that can be obtained from the excretion of yew tree needles. Unlike other taxanes, cabazitaxel can overcome the increased expression of the multidrug resistance gene (MDR) in prostate cancer [66]. Compared to first-generation taxanes, paclitaxel and docetaxel, cabazitaxel has extra methyl groups that result in a lower affinity to the MDR’s p-glycoprotein or p-gp efflux pump. Moreover, cabazitaxel has high hydrophobicity and poor aqueous colloidal stability, which presents researchers with the opportunity to synthesize new cabazitaxel formulations with improved features [67].

##### Vinorelbine and Vindesine (Vinca Alkaloids)

Vinorelbine and vindesine are vinca alkaloids, which means they can act as microtubule inhibitors by inhibiting the polymerization of tubulin [31]. Like their first-generation predecessors, vincristine and vinblastine, vinorlbine and vindesine can act as anticancer agents. Vinorelbine may cause mild to moderate neuropathy, constipation, and nausea; however, it can help treat non-small cell lung cancer. Vindesine can cause bone marrow toxicity and has low renal excretion.

##### Ixabepilone

Ixabepilone is a semi-synthetic analog of epothilone B. Preclinical and clinical data have established the drug as a viable therapeutic option for the treatment of locally advanced or metastatic breast cancer where treatments with vinca alkaloids and taxanes were unsuccessful. Considering that 90% of people with metastatic cancer experience anticancer treatment failure, the emergence of ixabepilone promises a stronger defense against cancer mortality rates. The drug was FDA-approved in 2007 and is meant to combat diseases that have grown resistant to other chemotherapies [68]. Thus, it provides breast cancer patients with an alternative treatment to a seemingly hopeless battle.

### 3.2. Synthetic Checkpoint Kinase 1 (CHK1) Inhibitors

So far, the major focus has been mitotic inhibitors that target microtubules and/or microtubule-associated proteins like kinesin. However, mitotic inhibitors are not simply limited to these drugs.

Unlike other mitotic inhibitors, CHK1 inhibitors do not bind to microtubules or microtubule-associated proteins to stop cancer proliferation. Instead, CHK1 inhibitors can stop mitosis by impairing DNA synthesis and increasing DNA damage by inhibiting the key regulatory DNA damage checkpoint CHK1. Moreover, CHK1 inhibitors can significantly enhance the efficiency of chemotherapy. For this reason, CHK1 inhibitors like LY2603618 are of great interest.

LY2603618 (also known as rabusertib, Table 1) is an ATP-competitive CHK1 inhibitor that can be used to treat cancer. A study conducted by Wang et al. tested the drug’s effectiveness against human lung cancer cells and found that it resulted in cell cycle arrest in the G2/M phase [69]. Not only that, but it inhibited CHK1 autophosphorylation and increased DNA damage. This suggests that LY2603618 in lung cancer cells involves the inhibition of CHK1 phosphorylation and the activation of the DNA damage response network [69].

Another related study found that LY2603618 induced apoptosis of cancerous cells via mitotic defects. Additionally, when the drug was taken in combination with pemetrexed, tumor growth was observed to be impeded [70]. Further trials focused on the overall response rate, safety, and the effect of non-small cell lung cancer patients’ bodies on LY2603618 and pemetrexed [71]. Patients were given 500 mg/m^2^ of pemetrexed on day 1 and 150 mg/m^2^ on day 2 every 21 days. Out of the fifty-five patients involved in the study, none of them experienced a complete response. Partial response was observed in 5 patients, while stable disease was observed in 20. In the end, results showed that the combination of LY2603618 and pemetrexed was favorable in terms of safety and pharmacokinetics. On the other hand, in terms of clinical activity, the combination of the two therapies was not significant [71]. Further studies are required to understand further the role LY2603618 can have, if any, when combined with certain antimetabolites.

### 3.3. Synthetic Kinase Inhibitors

For cell division to be successful, protein kinases are used to regulate cell replication. The dysregulation of these enzymes has been found to correlate with various human cancers. Hence, inhibiting these essential kinases can lead to improved anticancer drug discovery as they can effectively inhibit tumor cell proliferation. Some of the best-known mitotic kinases are aurora kinases and polo-like kinases. These enzymes and their corresponding inhibitors are discussed in the following section.

#### 3.3.1. Aurora Kinase Inhibitors

As previously stated, aurora kinase enzymes are often overexpressed in tumor cells, implying that their inhibition could have some anticancer activity.

The aurora kinase family comprises Ser/Thr kinases and is actively involved in a wide multitude of cellular processes, including mitotic entry and cytokinesis. In eukaryotic cells, aurora A kinases (AKA) are responsible for mitotic spindle poles, mitotic entry, centrosome maturation/separation, and spindle bipolarity [72]. On the other hand, aurora B (AKB) of eukaryotes is involved in regulating chromosome-microtubule interactions, spindle stability, and cytokinesis [72].

Notable AKA inhibitors include alisertib (MLN8237) and ENMD-2076. These cancer treatments have already advanced through and passed human clinical trials. A study focusing on the effect of alisertib on human tumor cell lines found that aurora A autophosphorylation (through pT288) was inhibited, and apoptosis and G2/M accumulation increased. Alisertib is anticancerous and highly cell-permeable, which means it can successfully enter tumor cells. The drug appears to be generally safe for human administration [73].

ENMD-2076 is a small-molecule kinase inhibitor that can disrupt tumor proliferation through either angiogenesis or cell cycle arrest. When the drug was tested on different human solid tumors and hematopoietic cancer cell lines, including breast, colon, melanoma, and leukemia, it was observed that it significantly inhibited in vivo tumor growth. The drug inhibited aurora A kinase as had been expected and proved successful in inhibiting the angiogenic tyrosine kinases VEGFR3/KDR and FGFR1 [74].

AKB inhibitors include ZM447439, AZD1152, and hesperadin. Hesperadin works by binding indolinone moiety to the catalytic cleft of active AKB. It causes abnormal mitosis and cytokinesis impairment [75]. Barasertib, or AZD1152, was derived from the optimization of ZM447439. In the prodrug form of the active drug ZM447439, basartib contains a phosphate group that helps it with its solubility and specific target action, resulting in significantly higher bioactivity. Over the years, the prodrug has proven itself a leading therapeutic molecule by inhibiting cell proliferation and increasing apoptosis in various tumors and AML cell lines [75].

Another set of aurora kinase inhibitors of interest is pan-aurora kinase inhibitors, which include PHA-739358, SNS-314, CYC116, and PF-03815735. Most notably, PHA-739358 (Table 1) has been investigated as a potential treatment for metastatic melanoma. This is of special interest because metastatic melanoma is a difficult cancer to treat because of its strong resistance against many traditional chemotherapies. The specific study showed that the drug inhibited cell proliferation and induced apoptosis in a time- and dose-dependent manner. Furthermore, the study demonstrated that PHA-739358 has definite potential as a therapeutic agent for melanoma [76].

#### 3.3.2. Polo-like Kinase Inhibitors

Polo-like kinase 1 (PLK1) is a significant mitotic protein that regulates G2/M transition and cytokinesis. Like aurora kinase proteins, it is often overexpressed in various human cancer cells, and its inhibition can result in tumor growth arrest. Some small-molecule protein inhibitors have been discovered recently, including BI 6727, NMS-P937, and GSK461364A. Studies on BI6727 showed that the inhibitor targeted the catalytic domain of PLK1, PLK2, and PLK3, depending on its concentrations. It also caused G2/M phase arrest and polo-like spindle-resemblance phenotype, which resulted in eventual cell death in a wide range of tumor cell lines in vitro [77].

The GSK461364A inhibitor also caused the emergence of a polo-like spindle and metaphase arrest, supporting the idea that the PLK1 inhibitor can work as a good anticancer agent [77]. Despite evidence showing significant tumor growth arrest under the treatment of PLK1 inhibitors, many of the active compounds have resulted in noteworthy side effects such as neutropenia and thrombocytopenia. For this reason, further research has been done on PLKi which has been observed to have a higher selectivity, stronger potency, and better absorption, distribution, metabolism, and elimination (ADME) properties. Its mechanism of inhibition involves “mitotic arrest, synthetic lethal interactions, and promotion of autophagy in center cells [78]”. Currently PLKi is being tested clinically and researched for even further development.

### 3.4. Antibody–Drug Conjugates (ADCs) with Mitotic Inhibitors as Payloads

The antitumor activity of small-molecule mitotic inhibitors warrants their continued and extensive study. Research data support their use as effective anticancer treatments for their significant therapeutic activity. However, as with all other anticancer agents, mitotic inhibitors come with the chance of several side effects, such as fever, hair loss, and/or anemia, and many others. Different toxicities may be observed while administering several therapeutically active agents because the human body is a complex machine. The body consists of many mechanisms working together to maintain various bodily functions. As such, an effect in one mechanism can have an undesired impact on many other interconnected mechanisms. Therefore, when a patient is given an anticancer treatment, their body may experience decreased cancer cells and undesired toxicity. To decrease the extent of an active agent’s side effects, its delivery mode can be manipulated to observe minimal undesired interactions. However, in the context of ADCs, the discussion is no longer confined to small molecules. Instead, it discusses small molecules conjugated to a large biomolecule (an antibody).

One such method involves the use of antibody–drug conjugates (ADCs). ADCs combine monoclonal antibodies with a cytotoxic payload through a linker. The antibodies used in ADCs are immunoglobulins that are useful in the human immune system because they identify and neutralize unwanted pathogens [79]. ADCs are useful in cancer treatments because they can identify and bind to antigens through their fragment antigen-binding (Fab) variable region. By taking advantage of an antibody’s ability to specifically bind to target tumor cells and a cytotoxic agent’s ability to kill those tumor cells, antibody–drug conjugates produce therapeutic effects with greater specific delivery and fewer side effects. The linker linking the antibody and cytotoxin can also manipulate the molecule’s polarity, thereby regulating the agent’s pharmacokinetics and pharmacodynamics [79].

Since anti-mitotic molecules can effectively induce cancer cell death, they can act successfully as cytotoxins in antibody–drug conjugates. Such ADCs commonly studied include the use of taxanes, vinca alkaloids, emtansine (DM-1), monomethyl auristatin E (MMAE), and monomethyl auristatin F (MMAF) as cytotoxic payloads.

#### 3.4.1. ADCs Using Taxanes

Although paclitaxel and docetaxel are important anti-mitotic cancer drugs today, they often come with undesirable side effects and drug resistance [80]. To overcome these drawbacks, taxane–monoclonal antibody immunoconjugates have emerged as promising molecules capable of reducing the taxane’s toxicity with their tumor-specific delivery.

Paclitaxel–antibody conjugates have been studied and determined to be more soluble than their individual components and, in an in vivo model of mice and xenografted tumors, successful in preventing tumor growth [81]. Multiple studies from the early 2000s have shown similar promising results. Paclitaxel has been conjugated with monoclonal antibodies Erbitux (C225), Sc7301, and trastuzumab. Sc-7301–paclitaxel and trastuzumab–(A-Z-CINN linker)–paclitaxel immunoconjugates have been identified as potential target agents against HER-2-positive breast tumor cells [82,83].

A drawback of paclitaxel-conjugated ADCs encountered over the years has been its occasionally unsatisfactory preclinical anticarcinogenic effects in vivo. It was hypothesized that the cause of such insufficient results was the simultaneous use of hydrophobic linkers with ultra-hydrophobic paclitaxel. To test this, a study investigated the therapeutic activity of paclitaxel-conjugated ADCs with hydrophilic linkers. Results were promising, showing that the molecules with hydrophilic linkers had a superior efficacy and safety profile in vitro and in vivo [84].

Aside from paclitaxel, docetaxel and other semi-synthetic taxane derivatives have been investigated for their ADC potential. In one specific study, for example, docetaxel was conjugated to cetuximab and panitumumab via a heterobifunctional cross-linker and investigated for its in vitro EGFR (epidermal growth factor)-specific cytotoxicity and in vivo anticancer effects. Results showed that the docetaxel ADC had greater EGFR-specific cytotoxicity as well as improved survival in treated mice, thus supporting the further study of taxane–monoclonal antibody immunoconjugates as effective cancer treatments with lower side effects [85].

#### 3.4.2. ADCs Using Vinca Alkaloids

Like taxanes, vinca alkaloids are important anti-mitotic anticancer agents that can cause patients to experience different side effects. Researchers have investigated monoclonal antibody–vinca alkaloid conjugates’ antitumor activity and safety profile to combat toxicity. Most of this research was done in the late 1990s, with only a few studies conducted in the 2000s.

In two separate studies, the therapeutic potential of the vinca alkaloid immunoconjugates LY256787 and LY203725 was tested and determined to be higher than that of free drug therapy [86]. The vinca alkaloid ADCs also improved efficacy and safety [87].

Monoclonal anti-carcinoembryonic–vinca alkaloid immunoconjugates have also been evaluated for their antitumor activity. When used to treat 10 human tumor cell lines, a study found that the conjugate’s efficacy was greatly correlated with its selectivity [88]. This is significant as it suggests that vinca alkaloid ADCs can successfully lower the toxicity of useful anti-mitotic inhibitors. To further understand the extent of vinca alkaloid ADCs, more current studies on its therapeutic effect and safety profile would be useful.

#### 3.4.3. ADCs Using DM-1

The ADCs of DM1, a cytotoxic anti-microtubule agent capable of inducing cell death, have shown significant anticancer activity. For instance, T-DM1 (trastuzumab emtansine) has demonstrated significant efficacy in treating metastatic diseases. As of 2019, around 100 clinical trials have been conducted on T-DM1, studying its role in HER2 malignancies, possible combinations with immunotherapy, and its function in metastasis [89]. Randomized trials have documented it as effective against advanced breast cancer in first-line, second-line, and after-second-line treatment [90].

Many factors have been identified as possible impairments to the cytotoxicity of T-DM1 in the treatment of HER2-positive metastatic breast cancer. These include inefficient internalization, impaired lysosomal degradation of trastuzumab, multidrug resistance proteins, enhanced recycling of the HER2–T-DM1 complex in cancer cells, or intracellular trafficking of HER2 [90]. Although primary resistance of HER2-positive metastatic breast cancer to ADC has been reported to be relatively infrequent, the majority of treated patients develop some drug resistance.

To improve T-DM1’s efficacy and thereby reduce patient resistance, the way the active agent facilitates its activity, as well as the resistance mechanisms of each of its mediating features, has been investigated. A recent 2020 study found that T-DM1 resistance mechanisms mostly correlate with dysfunctional intracellular metabolism of DM1-mediated cell killing construction and subversion. From this data, strategies for combating T-DM1 resistance can be further constructed, such as using alternative linker–payload chemistries [91].

#### 3.4.4. ADCs Using MMAE

Monomethyl auristatin E (MMAE) is a potent mitotic inhibitor that can induce cancer cell death by blocking tubulin polymerization. Because of its high cytotoxicity, MMAE cannot be used as a treatment drug by itself; instead, it can be administered with antibody–drug conjugates by linking it with cleavable linkers to monoclonal antibodies that can increase its specificity and reduce toxicity towards healthy cells (side effects) [92].

An example of an MMAE-containing ADC is polatuzumab-vedotin. This anticancer agent comprises an anti-CD79B IgG1 monoclonal antibody linked to MMAE. Before the molecule was allowed human clinical trials, it was tested on monkeys. Results showed that the ADC’s MMAE-driven myotoxicity had good antitumor activity and good enough pharmacokinetic/pharmacodynamic properties for clinical trials [93]. Hence, polatuzumab-vedotin is a good option for administering MMAE while avoiding its high cytotoxicity, which negatively impacts the body.

M69-MMAE is another MMAE-conjugated ADC of interest. This molecule has a novel antibody that targets matriptase, a transmembrane serine protease and cell-surface enzyme that plays an important role in tumor initiation and progression, via a valine-citrulline-PABA linker [94]. M69-MMAE has been found to be effective against triple-negative breast cancer cell lines and xenografts. Future endeavors include obtaining additional preclinical data of the ADC’s chemotherapeutic activity alone and in combination with other anticancer drugs to allow for future clinical development.

#### 3.4.5. ADCs Using MMAF

Like its MMAE analog, MMAF (monomethyl auristatin F) can also act as a mitotic inhibitor by blocking tubulin polymerization. Since it is so highly toxic, it cannot act as a drug on its own but can induce antitumor effects when administered through an ADC.

A 2021 study on a new linker system found that its MMAF-conjugated ADCs, called LegoChem Bisciences-ADC (LCB-ADC), displayed higher cytotoxicity than T-DM1 by noticeably inhibiting tumor growth in a HER2-high-expressing N87 xenograft tumor [95]. Additionally, it was determined that LCB-ADC has higher efficacy and biostability than its ADC counterparts. These results suggest that this anticancer agent, with its MMAF cytotoxin and elaborate linker, can successfully treat cancers that have been difficult to treat with prior therapeutic molecules.

### 3.5. Clinical Studies

**Figure 2:**
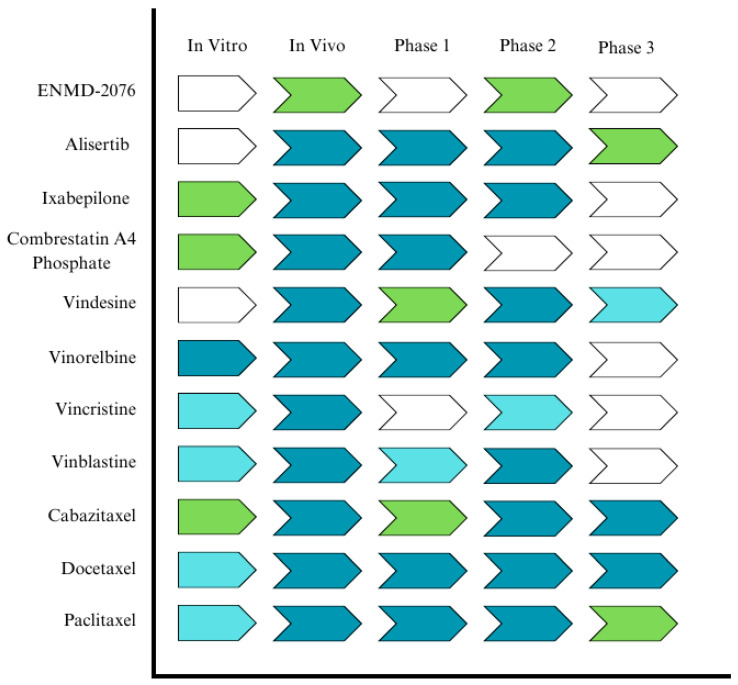
Landscape design of available data for small-molecule mitotic inhibitors as anticancer agents. White = no data available, Green = data available from one single study, Light Blue = data available from two to three studies, Dark Blue = data available from more than three studies.

Clinical trial studies on the anti-mitotic agents and subsequent information obtained from in vitro, in vivo, phase 1, phase 2, and/or phase 3 trials are schematically presented in Figure 2. For these studies, it is important to mention that cancer cells in the human body differ significantly from those cultured in labs, often having longer doubling times in anti-mitotic chemotherapy. This could possibly be related to why inhibitors of kinesin-5 and aurora kinase have not been approved as anticancer drugs despite their good to excellent anticancer activity. As the main focus of this section is to review clinical studies for mitotic inhibitors only, accordingly, their synergetic effects when administered with other therapeutic agents have not been included in this review. In the United States, when phase III clinical trials (in a few cases, phase II trials) show a new drug is more effective or safer than the current treatment, a new drug application (NDA) is submitted to the Food and Drug Administration (FDA) for approval. Accordingly, in Figure 2, the inhibitors that did not enter Phase III trials are not eligible for FDA approval.

#### 3.5.1. Paclitaxel

The most recent in vivo study investigated the drug’s in vivo inhibition potency against membrane proteins OATP1B1 and OATP1B3 using endogenous OATP1B biomarkers [96]. Ten patients with non-small cell lung cancer were administered 200 mg/m^2^ of paclitaxel via a 3-h infusion. It was found that the mitotic inhibitor significantly inhibited OATP1BI during and at the end of the infusion.

Both in vitro and in vivo tumor models showed that paclitaxel promotes the polymerization of tubulin and inhibits depolymerization; however, it is not as potent as docetaxel [97]. In another study, the drug’s pharmacokinetic characteristics showed that the agent had extensive tissue distribution, high plasma protein binding, and minimal renal excretion of the parent drug (less than 10%) [98].

Paclitaxel has been used in Phase 1 studies (NCT03246074, completed in 2024) against ovarian cancer, advanced solid tumors, acute leukemias in pediatric patients, and cancer in patients with severe hepatic dysfunction [99,100,101,102]. In these studies, the drug’s toxicity and pharmacokinetics were investigated, and it was found that toxicity was schedule-dependent.

In Phase 2 clinical trials, the drug was used on patients with relapsed and refractory small cell lung cancer (SCLC) as well as patients with non-small cell lung cancer (NSCLC). It was found that paclitaxel was effective in treating refractory SCLC when administered weekly with 80 mg/m^2^ infusions. Toxicities included infection, skin rash, neuropathy, and pulmonary toxicity [103]. Toxicities for NSCLC most commonly observed were grade 3/4 leukopenia and grade 4 neutropenia. Other toxic effects included fever, arthralgia, and myalgia [104]. An ongoing randomized Phase III trial (NCT05116189) compares paclitaxel/carboplatin/maintenance letrozole with letrozole monotherapy in patients with advanced or recurrent endometrial cancer.

Beyond SCLC and NSCLS, Phase 2 clinical trials of paclitaxel investigated its effect on gastric carcinoma (NCT00855764, completed in 2010), advanced squamous cell penile cancer, advanced breast cancer in Japan, and unresectable hepatocellular carcinoma patients. Data suggested that paclitaxel has partial activity against gastric carcinoma, and its combination with other active agents may be effective in combating cancer [105]. Regarding the agent’s effect on advanced breast cancer in Japanese patients, it was found that it had a relatively high response against the disease, making it a good candidate for further research [106].

#### 3.5.2. Docetaxel

Multiple Phase 1 studies have been reported for docetaxel, especially those about the drug’s maximum tolerated dose (MTD) and toxic effects. One such study investigated the drug’s MTD and basic pharmacokinetics in the day-1 and -8 schedule by treating 32 patients with refractory solid malignancies with a 1-h infusion of docetaxel on a day-1 and -8 schedule. The main toxicities were found to be neutropenia, asthenia, alopecia, and hypersensitivity reactions, but the drug showed promising activity in patients with refractory breast and ovarian neoplasms [107]. Another study found that weekly docetaxel administration on pretreated metastatic breast cancer patients was not as promising because it caused the progressive emergence of nonhematological side effects despite noticeably higher activity [108].

In Phase 2 studies, docetaxel had little activity against metastatic colorectal carcinomas but had significant clinical activity against untreated and pretreated NSCLC, along with acceptable toxicity [109].

During a randomized Phase 2/3 study, docetaxel was used as a radiosensitizer for cisplatin-ineligible patients with locally advanced head and neck squamous cell carcinoma (LAHNSCC). The median overall survival (OS) of patients administered docetaxel was 25.5 months, which could be considered a significant improvement from an OS of 15.3 months for patients without docetaxel. From this study, it can be concluded that docetaxel has the potential to act as a good radiosensitizer for LAHNSCC patients (completed in 2023, [110]).

#### 3.5.3. Cabazitaxel

Although limited in vitro data are available on cabazitaxel, various Phase 2 and Phase 3 clinical studies have been conducted on it. A Phase 1 study investigated the drug’s pharmacokinetic characteristics and antitumor activity in a large population of Japanese patients with metastatic castration-resistant prostate cancer (mCRPC). Patients treated with a maximum tolerated dose of 25 mg/m^2^ every 3 weeks experienced adverse effects such as neutropenia (100%), fatigue (54.5%), nausea (52.3%), and diarrhea (50.0%). Out of the 12 patients in the efficacy population, two had a partial response while the rest had stable disease [111]. These data suggests that, although cabazitaxel had consistent efficacy in a large population, further toxicity management is needed to combat its significant side effects.

For Phase 2 and 3 studies, cabazitaxel was compared to docetaxel. This is in line with cabazitaxel’s structure, which allows it to be effective in some cases where docetaxel chemotherapy was unsuccessful. In a 2016 Phase 2 study, cabazitaxel was administered to patients with advanced NSCLC progressing under or after docetaxel treatments. Data suggested that despite a substantial toxicity profile, the drug exhibited activity against pre-treated NSCLC [112]. Moreover, a Phase 3 clinical trial found that cabazitaxel significantly improved the overall survival (OS) of mCRCP patients who had been treated with docetaxel (NCT01308567, [113]). All this data further supports the continuous research on cabazitaxel as an alternative anticancer treatment after other treatments have failed.

#### 3.5.4. Vinblastine

Vinblastine has been recorded to have potent antiproliferative effects in vitro against canine transitional cell carcinoma (TCC) [114]. Additionally, in another in vitro study, vinblastine was determined to induce CYP3A4 via an NR1I2-dependent mechanism [115].

In vivo studies have researched the effect of vinblastine on dogs and patients with advanced cancer, MRCC, or ovarian carcinoma. A Phase 2 clinical trial also investigated the use of vinblastine with radiochemotherapy against invasive bladder cancer. Of the 84 bladder cancer patients treated, it was observed to be effective in more than half of the group. A downside to the treatment, however, was impaired bladder function, which suggests that further research is needed on reducing chronic morbidity [116].

#### 3.5.5. Vincristine

Although well over a thousand studies have been conducted using vincristine, most have been done in combination with other anticancer drugs, such as cyclophosphamide (CP). In other words, many clinical studies with vincristine are not standalone. Accordingly, the studies where vincristine was combined with other cytotoxic agents are beyond this paper’s scope.

Nonetheless, a handful of in vitro and Phase 2 studies are still available for review. In vitro studies have shown that vincristine’s cytotoxic activity is correlated with extracellular concentration and the duration of exposure [117]. Extracellular concentration, however, is limited by neurotoxicity concerns. To address this drawback, in vivo studies have investigated alternative ways of enhancing vincristine’s efficacy—one such way being prolonging the duration of in vivo exposure. A clinical trial investigated the neurotoxicity and pharmacokinetics of vincristine when it was administered to 16 patients (children with brain tumors) via a 96-h continuous infusion after a conventional bolus dose. Patients reported a wide range of side effects, such as jaw pain, constipation, and mild abdominal pain [118]. However, there was no Grade IV toxicity and only one Grade III toxicity observed. Moreover, a complete response was monitored in one patient, a partial response in three patients, and stable disease in seven patients. Disease progression was observed in the remaining three patients. From this study, it was determined that continuous infusion of vincristine after a conventional bolus dose could effectively act as a safe way of increasing systemic exposure without exposing children with tumors of the central nervous system to significant neurotoxicity. This is significant because it shows that increasing in vivo exposure can effectively combat concerns about vincristine toxicity.

Beyond children with brain tumors, vincristine has also been found to be active against some cases of non-Hodgkin’s lymphoma, advanced/recurrent endometrial carcinoma, and non-small bronchogenic carcinoma in Phase 2 clinical studies. Regarding advanced/recurrent endometrial carcinoma, however, it was also found that it resulted in significant toxicity when 1.4 mg/m^2^ of it was administered weekly to patients via intravenous bolus for 4 weeks and then every other week [119]. As such, this study shows just how much of a role the anticancer agent’s dose and schedule plays in its toxicity.

#### 3.5.6. Vinorelbine

In vitro and in vivo preclinical studies have implied that vinorelbine is active against SCLC and NSCLC. Additionally, many in vitro studies have suggested that vinorelbine and paclitaxel have synergetic activity [120]. A Phase 1 study and multiple Phase 2 trials have reported that the anticancer agents work well in combination to treat patients with extensive-stage small cell lung cancer.

Vinorelbine has also been found to act as a strong radiosensitizer in vitro and a Phase 1 trial in locally advanced non-small cell lung cancer [121]. However, more data from future Phase 2 trials are needed on the recommended daily dose of vinorelbine for concurrent thoracic radiotherapy.

Vinorelbine has been tested in both dogs and cats. For example, in a Phase 1 trial, vinorelbine (VRL) was administered 61 times to 19 cats. Acute dose-limiting toxicities (DLTs) were observed to include neutropenia, vomiting, and nephrotoxicity [122]. It was determined that VRL was tolerated in cats at a weekly interval, but further investigation of its efficacy in treating malignancies is needed.

In other Phase 1 studies, metronomic oral vinorelbine has been administered to human patients with advanced cancer and metastatic NSCLC. Such trials found that oral VRL is effective when given to patients with advanced cancer via a metronomic schedule of 50 mg thrice weekly for 3 out of 4 weeks with minimal toxicity [123]. The difference in activity of oral and intravenous VRL has also been widely clinically studied in both Phase 1 and Phase 2 trials. A randomized Phase 2 trial investigated the efficacy and safety of oral vinorelbine versus intravenous vinorelbine in patients with NSCLC. It determined that the two administrations had comparable activity with qualitatively similar safety profiles [124]. In other words, oral VRL is a good alternative to intravenous administration, which is favorable because it provides patients with more variety depending on their needs. Other Phase 2 trials have used VRL to treat patients with advanced gastroesophageal adenocarcinoma, metastatic and advanced breast cancer, relapsed ovarian cancer, metastatic squamous cell carcinoma of the esophagus and head/neck, metastatic prostatic carcinoma, advanced and/or recurrent cervical carcinoma, and inoperable non-small cell lung carcinoma [125,126,127,128,129,130,131,132,133].

#### 3.5.7. Vindesine

Over a hundred clinical studies have been conducted using vindesine, yet not much in vitro data has been found readily available. Most data for this semi-synthetic vinca alkaloid have been derived from various Phase 2 trials.

Two separate Phase 2 studies investigated the treatment of hematologic malignancies with vindesine [134,135]. In one study, patients with hematologic malignancies refractory to conventional chemotherapy experienced a mix of complete and partial remissions, with responses depending greatly on the frequency of their treatment schedule. Additionally, although neurotoxicity was observed, it was generally mild in degree and infrequent. Similar results were observed in the other study; however, in that case, two patients with Burkitt’s lymphoma were discontinued from the treatment because of significant neurotoxicity, such as abdominal distension and severe constipation. Other Phase 2 trials have studied vindesine’s treatment of malignant tumors and hepatocellular cancer as well as its effect on children with leukemia and lymphoma. It was determined that while vindesine does not have a therapeutic effect in patients with hepatocellular carcinoma, it does have antitumor activity in patients with leukemia, lymphoma, and testicular neoplasms. However, vindesine administration may cause birth defects and may increase infection incidence and neuropathy; therefore, considering the serious risk factors, further trials are not encouraged [136,137].

#### 3.5.8. Combretastatin A4 Phosphate

Combretastatin A4 phosphate (CA-4P) has been shown to exhibit toxicity toward proliferating endothelial cells in vitro [138]. Although the tubulin-binding agent has been shown to destroy tumor blood vessels and act successfully as a targeting anticancer drug in animal models and clinical trials, it has adverse effects (NCT00395434, [139]).

Phase 1 studies testing CA-4P’s tolerability have found that it is consistent with other ‘vascularly active’ drugs but demonstrated notable side effects, and the trial was discontinued [140].

#### 3.5.9. Ixabepilone

Several clinical trials have been conducted on the epothilone B analog ixabepilone to investigate its anticancer activity in a wide range of patients. Phase 1 studies have supported in vitro pharmacodynamic observations of ixabepilone in which the anticancer agent has been observed to cause microtubule bundle formation in tumor cells, resulting in eventual cell death [141]. Most phase 1 studies investigated ixabepilone’s maximum tolerated dose (oral and intravenous), dose-limiting toxicities, safety profile, pharmacokinetics, and anticancer activity [142]. Common dose-limiting toxicities have included neutropenia, stomatitis/pharyngitis, myalgia, and arthralgia, while the maximum tolerated dose at 1-h infusion every 3 weeks was determined to be 50 mg/m^2^. The effect of ixabepilone on children, adolescents, and adults with solid tumors has also been studied, and it has been shown that the agent has good anticancer activity and tolerability [143]. Further research on ixabepilone’s single and combination therapeutic activity continues to be interesting.

Beyond Phase 1 trials, multiple Phase 2 clinical trials have tested ixabepilone for its treatment of patients with cancers that were resistant to anthracycline, taxane, and/or capecitabine or with tumors that had previously failed treatment with platinum-based chemotherapy [144,145,146]. These data support the use of ixabepilone as an alternative cancer treatment when other more common cancer treatments have failed.

Phase 2 trials have also determined that ixabepilone has limited activity in treating advanced hepatobiliary cancers and no meaningful activity in treating patients with metastatic melanoma [147,148]. It did, however, have significant anticancer activity in treating men with metastatic castrate-resistant prostate cancer, women with metastatic breast cancer previously untreated with taxanes, and patients with advanced pancreatic cancer [149,150,151].

#### 3.5.10. Alisertib (MLN8237)

In 2014, a Phase 1 study of alisertib investigated the therapeutic activity of the aurora A kinase inhibitor against relapsed/refractory multiple myeloma, non-Hodgkin lymphoma, and chronic lymphocytic leukemia. In the study, six out of the fifty-eight patients administered MLN8237 orally experienced a partial response, whereas thirteen achieved stable disease (NCT01482962, [152]). Observed drug-related toxicities included neutropenia, thrombocytopenia, anemia, and leukopenia.

Alisertib’s treatment of advanced solid tumors, safety, pharmacokinetics, pharmacodynamics, and the bioavailability of two oral formulations have also been studied through other Phase 1 trials. One such trial concluded that MLN8237 had sufficient tolerability and favorable pharmacokinetics in a sample population of 87 adult patients with advanced solid tumors [153]. Additionally, it was determined that the drug’s maximum tolerated dose is 50 mg twice a day for 7 days in 21-day cycles. This data established a recommended dose for Phase 2 trials involving the treatment of various solid tumors and hematologic malignancies and a recommended dose for a Phase 3 trial with peripheral T-cell lymphoma [154].

The effects of alisertib on patients with platinum-resistant/refractory epithelial ovarian carcinoma, fallopian tube carcinoma, primary peritoneal carcinoma, salvage malignant mesothelioma, and recurrent/refractory solid tumors or leukemia have been studied in Phase 2 trials. It was found that MLN8237 has modest antitumor activity and may produce responses in some patients with platinum-resistant ovarian cancer or malignant mesothelioma, as reported in 2016 ([155], NCT02293005, [156]). On the other hand, when looking at the effect on children and adolescents with recurrent/refractory solid tumors or leukemia, a low response rate of less than 5% was observed (NCT01154816, [157]). In other words, MLN8237 may be a suitable option for treating certain cancers in adults, but not in pediatric ones.

#### 3.5.11. ENMD-2076

The inhibitor ENMD-2076 has been studied for its safety and activity against platinum-resistant recurrent epithelial ovarian cancer in a Phase 2 trial from 2013 [158]. A total of 64 patients were administered ENMD-2076 daily via an oral dose. Some recorded adverse effects were fatigue, hypertension, and diarrhea. In the end, it was concluded that ENMD-2076, and further research on the inhibitor, is of interest for its anticancer activity against platinum-resistant ovarian cancer and tolerable toxicity [159].

## 4. Conclusions and Future Aspects

With increasing technological advancements and continuous research, cancer-related deaths in the U.S. have significantly decreased over recent years. From the many cancer treatments applied, small-molecule mitotic inhibitors stand out for their cytotoxicity and diversity. This review provides a comprehensive overview of available mitotic inhibitors from both natural and non-natural sources, a summary of their recent developments, and a discussion of their future prospects. Small-molecule mitotic inhibitors were discussed and classified through their discovery, anticancer activity, and mechanisms of action. With this, mitotic inhibitors are further confirmed as effective treatments for various types of cancers. However, it has also been found that their application can result in a wide range of side effects. To increase potency and reduce toxicity via targeted therapy, antibody–drug conjugates and prospective derivatives of known mitotic inhibitors are discussed as plausible ways of increasing target specificity while still maintaining anticancer activity. Although cancer can be a difficult disease to treat because of its enhanced proliferation, mitotic inhibitors provide researchers and physicians with different ways to stop the growth and spread of tumor cells by inhibiting mitosis. Some drugs can do this by disrupting the cells’ microtubules or microtubule-regulating proteins, such as the kinesin-inhibiting drug monastrol. Others do so by inhibiting the kinase checkpoint protein, CHK1, or kinase mitotic aurora proteins. Regardless of the specific method, it is clear from this account that a variety of synthetic, semi-synthetic, and natural molecules can be used to combat cancer through mitotic inhibition. This shows that future research and development of these small molecules can potentially decrease cancer mortality in the U.S.

Some aspects to consider for future research are different ways of reducing drug toxicity as well as increasing potency. Drugs under the taxane and vinca alkaloid families have shown significant anticancer activity; however, they have also been observed to cause a handful of side effects, such as nausea, muscle pain, and neuropathy. For this reason, more research is needed on increasing the drugs’ target specificity to reduce side effects. One such way of targeted therapy might be the use of antibody–drug conjugates. Another way is by researching analogs or derivatives of anticancer mitotic inhibitors to increase bioavailability and target specificity. Targeted drug delivery can be a third option also. For instance, blebbistatin has shown significant anticancer activity with poor bioavailability. However, its derivatives have shown better solubility and target specificity, decreasing the extent of their side effects. Therefore, derivatives of mitotic inhibitors like blebbistatin can be used to help maintain antineoplastic action while at the same time increasing delivery specificity. Moreover, extensive research on recently developed target-specific mitotic inhibitors (e.g., kinesin-5 inhibitors, Plk1 inhibitors, aurora inhibitors) may potentially contribute to future anti-mitotic chemotherapy.

## 5. Abbreviations

Microtubule-Associated Proteins (MAPs); Copy Number Alterations (CNAs); Polo-like Kinase 1 (PLK1); Microtubules (MT); Nordihydroguaiaretic Acid (NDGA); Estramustine (Emcyt); Bcl-2-Associated Death Promoter (BAD); Stilbenes (combretastatin A); Dihydrostilbenes (combretastatin B); Phenanthrenes (combretastatin C); Macrocyclic Lactones (combretastatin D); Histone Deacetylase Activity (HDAC); Trichostatin A (TSA); Heat Shock Protein 70 (HSP 70); N-terminal Nucleotide-binding Domain (NBD); Dihydropyrimidine (DHPM); S-Trityl-L-Cysteine (STLC); Hedgehog (Hh); Smoothened (Smo); Multidrug Resistance Gene (MDR); Checkpoint Kinase 1 (Chk 1); Aurora A Kinase (AKA); Aurora B Kinase (AKB); Alisertib (MLN8237); Small Cell Lung Cancer (SCLC); Non-Small Cell Lung Cancer (NSCLC); Maximum Tolerated Dose (MTD); Locally Advanced Head and Neck Squamous Cell Carcinoma (LAHNSCC); Overall Survival (OS); Metastatic Castration-Resistant Prostate Cancer (mCRPC); Transitional Cell Carcinoma (TCC); Cyclophosphamide (CP); Vinorelbine (VRL); Dose-Limiting Toxicities (DLTs); Combretastatin A4 Phosphate (CA-4P); Combretastatin A4 (CA-4), Antibody–Drug Conjugates (ADCs), Fragment Antigen-Binding (Fab), Emtansine (DM-1) Monomethyl Auristatin E (MMAE), and Monomethyl Auristatin F (MMAF), EGFR (epidermal growth factor), LegoChem Bisciences-ADC (LCB-ADC), T-DM1 (Trastuzumab Emtansine).

## Figures and Tables

**Table 1:** Classification and structure of different microtubule(-protein) binding drugs [22].

Compound (Class)	Mechanisms of Action	Structure and Stereochemistry
Paclitaxel (a taxane) (Natural)	Stabilizes microtubules and disrupts normal spindle dynamics during cell division	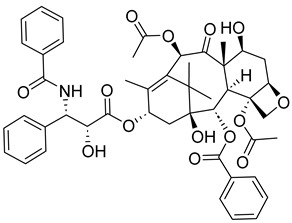
Docetaxel (Semi-Synthetic)	Interacts with microtubules to disrupt regular cell division	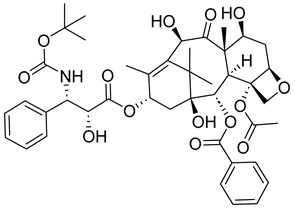
Cabazitaxel (Semi-Synthetic)	Interacts with microtubules to disrupt regular cell division	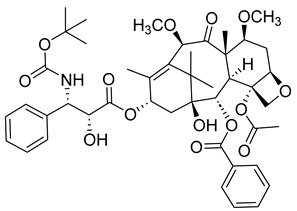
Vinblastine (Natural)	Prevents proper mitotic spindle formation and chromosome segregation	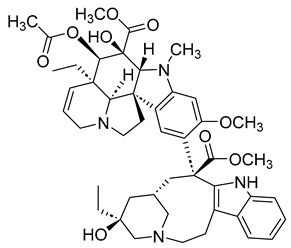
Vinorelbine (Semi-Synthetic)	Disrupts microtubule dynamics and interphases apoptosis induction	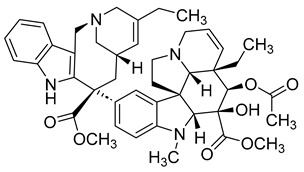
Vindesine (Semi-Synthetic)	Disrupts microtubule dynamics with distinct concentration-dependent effects and downstream consequences for mitotic progression	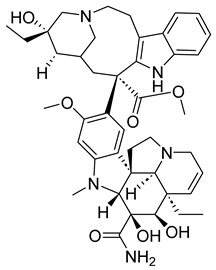
Vincristine (Semi-Synthetic)	Disrupts microtubule dynamics, leading to mitotic arrest via spindle assembly checkpoint activation	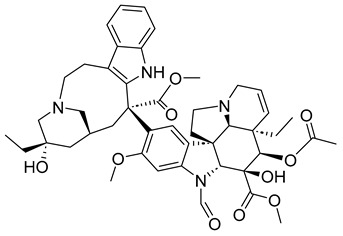
Epothilone (Natural)	Stabilizes microtubules and disrupts their dynamic behavior	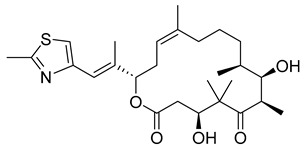
Nocodazole (Synthetic)	Inhibits cellular division by disrupting microtubule dynamics	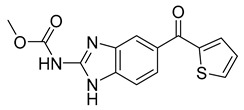
Combretastatin A4 (Natural)	Interacts with tubulin and subsequently disrupts the microtubule dynamics	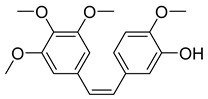
Trichostatin A (Natural)	Acts as an HDAC (histone deacetylase) inhibitor, leading to several downstream effects on cell cycle regulation and mitotic progression	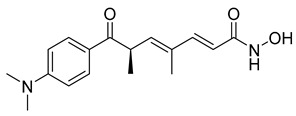
Monastrol (Synthetic)	Inhibits the mitotic kinesin Eg5 (*aka* KIF11 or kinesin-5)	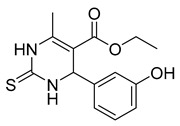
Monastrol Analog-1 (MA-1) (Synthetic)	Inhibits the mitotic kinesin Eg5 (*aka* KIF11 or kinesin-5)	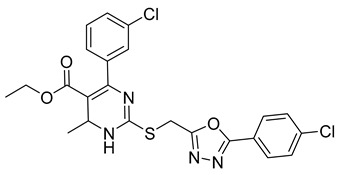
Monastrol Analog-2 (MA-2) (Synthetic)	Inhibits the mitotic kinesin Eg5 (*aka* KIF11 or kinesin-5)	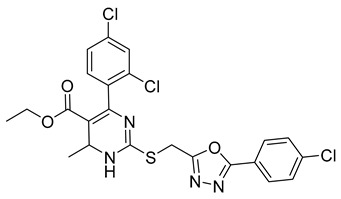
Dimethylenastron (a monastrol analog), (Synthetic)	Binds to a specific allosteric site on the Eg5 motor domain near the ATP/ADP binding pocket	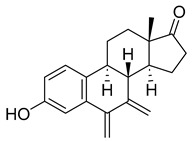
S-Trytyl-L-Cysteine (STLC) (Synthetic)	Binds to a specific allosteric site on the Eg5 motor domain, positioned between helix three and loop 5	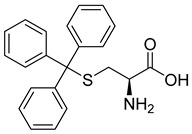
Dynarrestin (Synthetic)	Inhibits cytoplasmic dynein 1 and 2	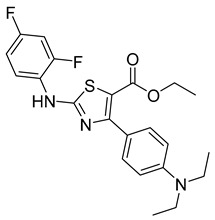
Nordihydroguaiaretic Acid (NDGA), (Natural)	Disrupts actin cytoskeleton activates stress-activated protein kinases, induces anoikis-like apoptosis, inhibits cyclin D1 and p300 acetyltransferase	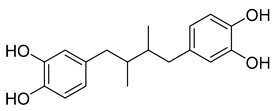
Blebbistatin (Synthetic)	Binds to an allosteric site on the myosin II motor domain, situated between the nucleotide pocket and the actin-binding cleft	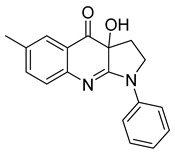
LY2603618 (Rabusertib) (Synthetic)	Inhibits checkpoint kinase 1 (Chk1)	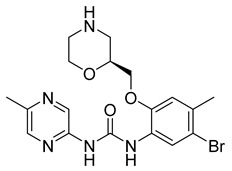
PHA-739358 (Danusertib) (Synthetic)	Inhibits aurora kinases	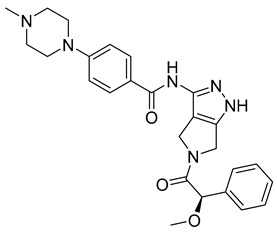
Alisertib (MLN8237) (Synthetic)	Inhibits aurora A kinase (AAK)	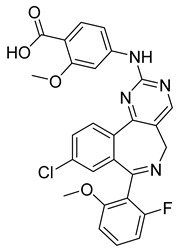
ENMD-2076 (Synthetic)	Inhibits AAK, which causes abnormal mitotic spindle formation, which leads to reduced spindle bipolarity and increases chromosome misalignment	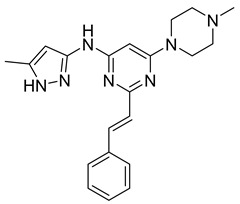
BI 6727 (Volaserib) (Synthetic)	Inhibits Polo-like kinase 1 (Plk1) that causes centrosome maturation, bipolar spindle formation, and chromosome alignment	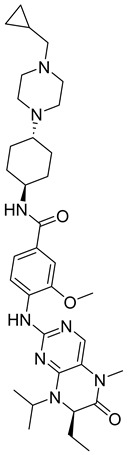
NMS-P937 (Onvansertib) (Synthetic)	Inhibits PLK1 and prevents multiple essential steps, including chromosome separation and cytokinesis	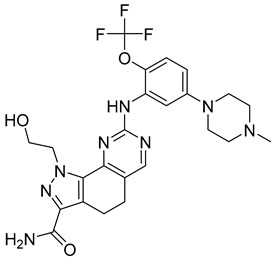
GSK461364A (Synthetic)	Inhibits PLK1 and ultimately leads to mitotic arrest at the G2/M phase of the cell cycle	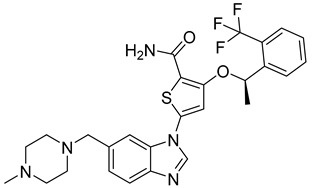
Ixabepilone (Semi-Synthetic)	Binds to β-tubulin subunits and suppresses their dynamic instability	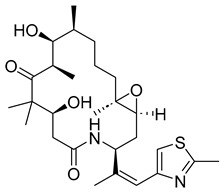
Colchicine (Natural)	Disrupts spindle formation and chromosome segregation during mitosis